# Bilateral Functional Electrical Stimulation for the Treatment of Presbyphonia in a Sheep Model

**DOI:** 10.1002/lary.30984

**Published:** 2023-08-19

**Authors:** Andrijana Kirsch, Claus Gerstenberger, Bernhard Jakubaß, Magdalena Tschernitz, Justin D. Perkins, Andrea Groselj‐Strele, Hermann Lanmüller, Jonathan C. Jarvis, Stefan Kniesburges, Michael Döllinger, Markus Gugatschka

**Affiliations:** ^1^ Division of Phoniatrics, ENT University Hospital Medical University of Graz Graz Austria; ^2^ Division of Phoniatrics and Pediatric Audiology at the Department of Otorhinolaryngology, Head and Neck Surgery University Hospital Erlangen, Friedrich‐Alexander‐Universität Erlangen‐Nürnberg Erlangen Germany; ^3^ Clinical Science and Services Royal Veterinary College Hatfield UK; ^4^ Core Facility Computational Bioanalytics, Center for Medical Research Medical University of Graz Graz Austria; ^5^ Center of Medical Physics and Biomedical Engineering Medical University of Vienna Vienna Austria; ^6^ School of Sport and Exercise Sciences Liverpool John Moores University Liverpool UK

**Keywords:** aged larynx, functional electrical stimulation, vocal fold atrophy

## Abstract

**Objectives:**

The aim of the study was to increase muscle volume and improve phonation characteristics of the aged ovine larynx by functional electrical stimulation (FES) using a minimally invasive surgical procedure.

**Methods:**

Stimulation electrodes were placed bilaterally near the terminal adduction branch of the recurrent laryngeal nerves (RLN). The electrodes were connected to battery powered pulse generators implanted subcutaneously at the neck region. Training patterns were programmed by an external programmer using a bidirectional radio frequency link. Training sessions were repeated automatically by the implant every other day for 1 week followed by every day for 8 weeks in the awake animal. Another group of animals were used as sham, with electrodes positioned but not connected to an implant. Outcome parameters included gene expression analysis, histological assessment of muscle fiber size, functional analysis, and volumetric measurements based on three‐dimensional reconstructions of the entire thyroarytenoid muscle (TAM).

**Results:**

Increase in minimal muscle fiber diameter and an improvement in vocal efficiency were observed following FES, compared with sham animals.

**Conclusion:**

This is the first study to demonstrate beneficial effects in the TAM of FES at molecular, histological, and functional levels. FES of the terminal branches of the RLN reversed the effects of age‐related changes and improved vocal efficiency.

**Level of Evidence:**

NA *Laryngoscope*, 134:848–854, 2024

## Graphical abstract

Bilateral functional electrical stimulation was applied to increase muscle volume and improve phonation characteristics of the aged sheep larynx. After 9 weeks of stimulation, an improvement in vocal efficiency as well as an increase in minimal muscle fiber diameter (type II fibers) was observed, compared with sham. Histology and gene expression analysis showed no significant muscle fiber type switching after stimulation.

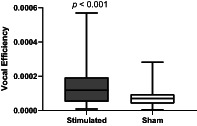

## INTRODUCTION

In a rapidly aging society, a steady increase in age‐related diseases is expected. Age‐related changes affect the larynx, with the incidence of impaired vocal function affecting 10–20% of elderly patients in modern Western societies, with a negative impact on quality of life.^1^ In patients with presbyphonia, age‐related atrophy of the laryngeal muscles, primarily the thyroarytenoid muscle (TAM), leads to vocal fold bowing and glottal gap.^2^, ^3^ This, combined with reduced laryngeal electromyographic amplitudes, leads to reduced sound pressure levels,^4^ resulting in a hoarse, breathy voice and significantly reduced vocal capacity. Current treatment is based on conservative speech therapy and laryngeal surgery (phonosurgery). Speech therapy is time‐ and cost‐consuming, whereas traditional phonosurgery only provides symptomatic therapy, without treating the underlying cause of presbyphonia.

In a previous study using a sheep model, we implanted an electrode using a minimally invasive surgical approach and showed that unilateral functional electrical stimulation (FES) of the terminal adductor branch of the recurrent laryngeal nerves (RLN) resulted in a significant volume increase of the stimulated TAM, compared with the unstimulated side.^5^ In the current study, we used the same approach bilaterally to investigate the functional consequences of TAM stimulation. We hypothesized that the increase in muscle volume would have an ameliorating effect on the phonatory characteristics of the aged sheep larynx.

## MATERIALS AND METHODS

### Animal Experiments

Female sheep aged approximately 10 years were used for the study. Given an average life expectancy of 11 years, and in line with similar studies, these sheep can be considered old.^6^ A total of 24 sheep were used, and they were assigned to two different cohorts (“functional/volumetric” and “molecular/histological,” 12 sheep each), based on the postexperimental analyses, as it was not possible to perform all analyses on the same larynges. Within each cohort, sheep were randomly assigned to either the stimulated group (*n* = 6) or the sham group (*n* = 6). All procedures were approved by the Austrian Federal Ministry of Education, Science and Research (BMBWF‐66.010/0015‐V/3b/2019), complied with the institution's animal care guidelines and were performed by experienced veterinarians. The skin was incised under general anesthesia,^7^ to expose the inferior margin of the thyroid cartilage. The tip of the stimulation electrode (K5‐P4, 5F, 4‐Pole Electrode, Osypka, Germany) was inserted using a custom‐made hollow needle (IT5F Split, Pajunk, Germany) that acted as a searching probe while connected to a stimulator (Pajunk, Germany). After identifying the “hot spot” (i.e., the position where a unilateral palpable muscle contraction of the TAM was elicited), the electrode was screwed into place. We sought to place the electrode near the cricothyroid joint, as the terminal branch of the RLN constantly passes this anatomical structure.^5^ The electrode was connected to the “MiniVstim 18B” implanted pulse generator (IPG) (improved version of a previously published model,^8^ Center for Medical Physics and Biomedical Engineering, Medical University of Vienna), which was placed subcutaneously in the neck region. This procedure was performed bilaterally. In the sham group, the electrodes were secured into the above‐mentioned position but not connected to the IPG. The IPG was programmed with predesigned training parameters via an external programmer using a bidirectional radio frequency link. A 9‐week long‐term stimulation protocol was started 1 week after implantation. Training sessions were automatically started by the IPG every other day in the first week of the training phase and every day for the following 8 weeks. The training duration was chosen to allow sufficient muscle growth.^9^ During the training phase, the terminal adductor branch of the RLN was stimulated at exactly the same time of day with 10 sets of contractions separated by 1 min. Each set consisted of 16 contractions (repetitions, 3 seconds on/0.5 seconds off). The stimulation frequency was 99 Hz, pulse width was 258 μs, and the initial amplitude was 0.5 to 3 mA. The total stimulation time was therefore 480 seconds per day, calculated from the “on” time in each set. The training pattern parameters were determined experimentally in previous studies.^5^, ^7^ The initial amplitude was adjusted for each sheep to three times the value that elicited the first detected response obtained endoscopically during the test stimulations. As simultaneous contraction of both left and right TAM could cause breathing problems, the stimulation of the left side was delayed (time shifted) compared with the right side: 24 h in the first week and 12 h in the following 8 weeks of the training phase. Once the IPG had been programmed, no further anesthesia or sedation was required. As in our previous study,^5^ the very first training session in awake animals was carefully monitored, and no signs of stress were observed. Every other week, we performed transnasal endoscopy in the sedated but awake animal to ensure the implant was functioning correctly by observing the VF movement produced in response to the stimulation.

### Tissue Harvest

After 9 weeks of training, animals were euthanized^7^ and their larynges harvested. For the “molecular/histological” cohort, the TAM (vocalis portion) and posterior cricoarytenoid muscle (PCAM) was harvested for histology and RNA isolation as previously described.^7^ Samples were stored at −80°C until processing. The TAM and PCAM were harvested bilaterally, except for two sheep in the cohort, in which the stimulation of the left side was not successful after 3 and 6 weeks, respectively. For these sheep, only the right stimulated side was harvested. For the “functional/volumetric cohort,” the larynges were shock frozen in liquid nitrogen and stored at −80°C until further processing.

### Functional Analysis

The multimodal measurement setup used in this study enables reproducible and highly accurate measurements of multiple aspects of the phonatory process within an ex vivo setting.^10^ The frozen harvested larynges were thawed overnight, prepared as previously described,^11^, ^12^ and mounted in the ex vivo setup in which measurements of the sound produced during phonation were made while adjusting air flow through the larynx and the loading of the vocal apparatus A detailed protocol is described in the Supplemental Information. In total, 768 measurement runs were performed. The analysis was performed in MATLAB version R2017b (The MathWorks, Inc, Natick, MA, USA). IBM SPSS software package version 28 (IBM, Armonk, NY, USA) was used for statistical analysis (Mann–Whitney *U* test).

### Micro Computed Tomography

After the completion of the functional analyses, the larynges were immersed in 4% phosphate‐buffered formalin solution for fixation and processed further as previously described,^11^ with minor modifications. Bruker SKYSCAN 1276 X‐Ray Microtomograph (Bruker Corporation, Billerica, Massachusetts, USA) with a maximum spatial resolution of 20 μm was used to generate CT images, whereas the scanning protocol was set using SKYSCAN 1276 measurement software. Volumes and diameter of the left and right TAM were averaged to obtain one value per sheep, except for one sheep, were a large sarcocystis was found within the right TAM. Statistical analysis was performed with SPSS (Mann–Whitney *U* test).

### Triple Immunofluorescence Labeling of Cryosections

Cryosections (10 μm) were prepared from frozen muscle samples and correct orientation of specimens was confirmed via standard haematoxylin and eosin staining. Subsequently, sections on Superfrost Plus microscope slides (Thermo Scientific, Waltham, MA, USA) were subjected to triple immunofluorescence labeling based on a previously described method.^7^, ^13^ A detailed protocol can be found in the Supplemental Information. In total, 34,558 fibers of the TAM were analyzed, 15,694 in the stimulated and 18,864 in the sham group. A total of 31,483 PCAM fibers were analyzed, 14,195 in the stimulated and 17,288 in the sham group. A Generalized Estimating Equations model^14^, ^15^ was used to compare treatment, fiber type, and minimum feret diameter using SPSS. GraphPad Prism 9.3.1. (San Diego, CA, USA) was used for the statistical analysis of muscle fiber type distribution (Mann–Whitney *U* test).

### Gene Expression Analysis

RNA isolation, reverse transcription, and quantitative polymerase chain reaction (RT‐qPCR) were performed as previously described.^7^ Primer sequences are provided in Table SI.

Each combination of cDNA sample and gene of interest was assayed in technical triplicates. Quantitation cycle (Cp) values from triplicates were averaged. Normalized relative quantities (NRQ) for all targets were calculated as previously described.^5^, ^7^ This strategy allows assessment of the relative abundances of distinct mRNAs within the same sample, as well as the relative comparison of mRNA levels between distinct samples. The NRQ values of the left and right side were averaged for every mRNA target (except in 2 sheep where only the right muscle was harvested), to obtain one value per target per sheep. Gene expression data were analyzed using GraphPad Prism (Mann–Whitney *U* test).

All figures were made using GraphPad Prism.

## RESULTS

All animals successfully completed the bilateral stimulation protocols except for two animals of the “histological/molecular group,” where it was unilateral. As these animals were not intended for functional analysis, the additional burden of a revision surgery was deemed unnecessary.

**Fig. 1:**
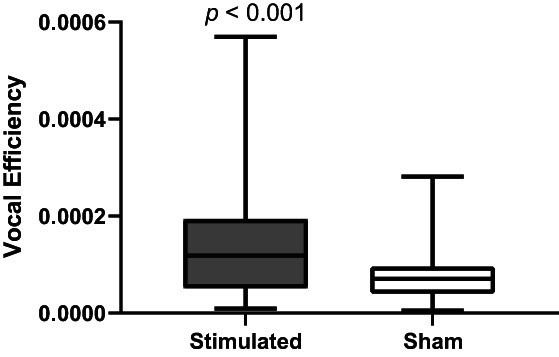
Box plot of vocal efficiency (VE) measurements.

VE data analysis was restricted to highly periodic vocal fold oscillations (defined as type 1 by Titze^16^). Data were also excluded if the video analysis failed because of bad contrast. Because of a large sarkocystis, one larynx of the sham group was excluded completely. Overall, 272 measurement runs (70.8%) from the sham group were included in the analysis and 376 (97.9%) of the stimulated group, which sums up to 648 (84.3%) of the total 768 recorded measurement runs.VE values of the stimulated group were significantly higher than those of the sham group (Mann–Whitney *U* test, *p* < 0.001, Fig. 1).

**Fig. 2:**
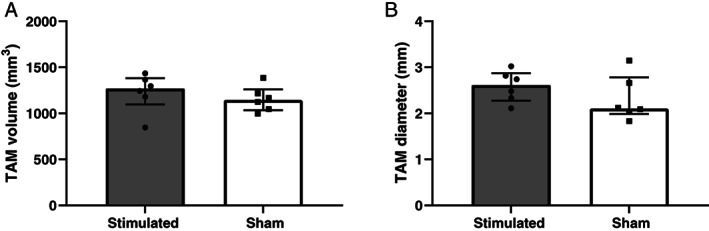
Thyroarytenoid muscle (TAM) volume (A) and diameter (B) analyzed with micro‐computed tomography (micro‐CT). Data are represented as median with interquartile range.

Mean volumes and diameters of the stimulated TAM were on average 6% and 16% larger than the sham group, respectively, but failed statistical significance (Fig. 2A,B, respectively).

**Fig. 3:**
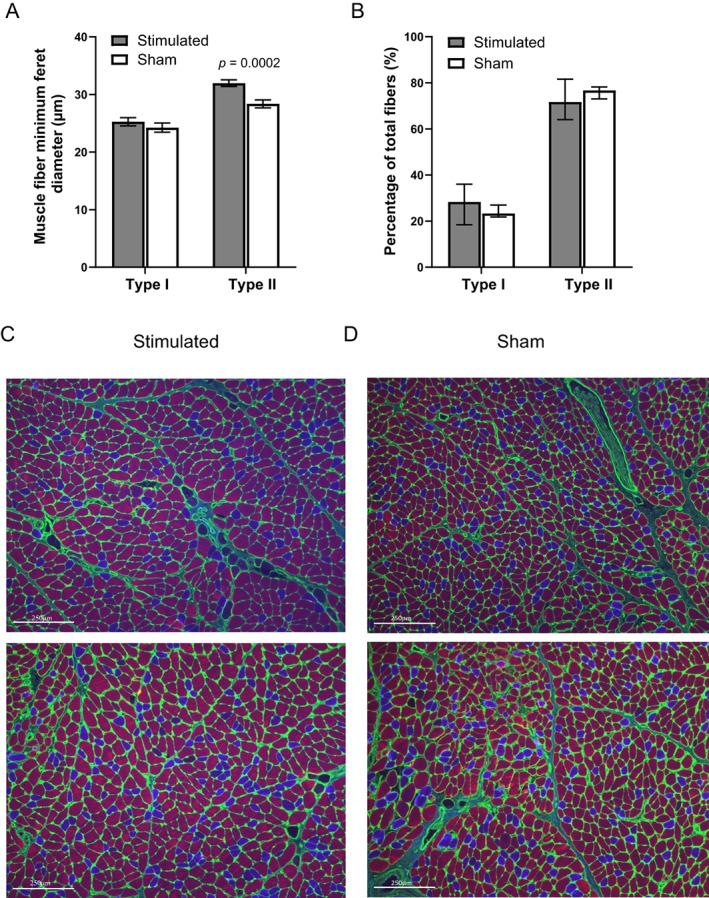
FES significantly increased minimum feret diameter of type II muscle fibers (A). Fiber‐type distribution was not altered (B). Representative images of stimulated (C) and sham (D) TAM. Green: collagen, blue: type I fibers, red: type II fiber, scale bar: 250 μm. Data are represented as mean with standard error (A) and median with interquartile range (B). [Color figure can be viewed in the online issue, which is available at www.laryngoscope.com.]

Immunohistology of the TA muscles revealed larger muscle fiber minimal feret diameter of type II but not type I fibers in the stimulated group, compared with the sham: 31.96 ± 0.57 versus 28.37 ± 0.69 μm (*p* = 0.0002), and 25.26 ± 0.69 versus 24.24 ± 0.79 μm, respectively (Fig. 3A). Fiber type distribution was not significantly altered in stimulated TAM, compared with sham (Fig. 3B). Representative images of stimulated and sham TAM are shown in Figure 3C,D, respectively.

**Fig. 4:**
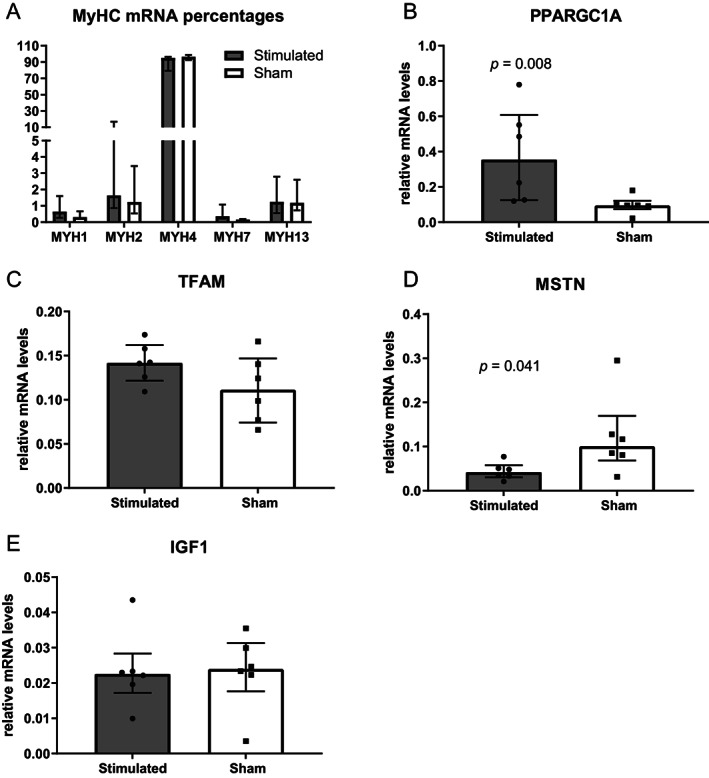
Reverse transcription, and quantitative polymerase chain reaction (RT‐qPCR) results. Myosin heavy chain (MyHC) isoform percentages (A), PPARGC1A: peroxisome proliferator‐activated receptor gamma coactivator 1‐alpha (B), TFAM: mitochondrial transcription factor A (C), MSTN: myostatin (D), and IGF1 = insulin‐like growth factor 1 (E). Data are represented as median with interquartile range.

Because excessive muscle contraction can induce a switch in muscle fiber types, which would impair TAM function, we investigated the expression of relevant sarcomeric myosin heavy chain (MyHC) isoforms. FES did not induce significant changes in the relative mRNA levels of any MyHC isoform (Fig. 4A). Gene expression analysis revealed that the expression of peroxisome proliferator‐activated receptor gamma coactivator 1‐α (PPARGC1A), a marker of mitochondriogenesis, was significantly upregulated in the stimulated group (*p* = 0.008, Fig. 4B). However, mitochondrial transcription factor A (TFAM), essential for the replication and translation of mitochondrial DNA, was not altered (Fig. 4C). Myostatin (MSTN), a muscle growth inhibiting cytokine, was downregulated in the stimulated group, compared with sham (*p* = 0.041, Fig. 4D), whereas insulin‐like growth factor 1 (IGF1) was unchanged (Fig. 4E).

As the RLN stimulation could affect the PCAM, we performed the same histological and gene expression analyses and found no significant changes in the minimal feret diameter or muscle fiber type distribution of stimulated PCAM, compared with sham (Fig. S1A,B, respectively). Likewise, no effect of the stimulation was seen on the gene expression level, compared with sham (Fig. S1C–G).

## DISCUSSION

We have previously shown that FES of the terminal (adduction) branches of the RLN resulted in a significant increase in the entire volume of the TAM muscle by 11%.^5^ However, only a unilateral stimulation protocol was used in this study. A bilateral stimulation protocol was needed to assess whether this increase in volume was sufficient to improve functional characteristics. Atrophy of the laryngeal muscles in presbyphonia leads to a glottal gap and reduced sound pressure levels,^2^, ^4^ resulting in a breathy voice and significantly reduced vocal capacity. VE, the ratio of acoustic power to aerodynamic power is decreased when the laryngeal mechanism is disordered.^17^ Glottal closure insufficiency and asymmetric VF oscillations decrease the VE value.^18^ Our study demonstrated for the first time that bilateral stimulation of the terminal adduction fibers of the RLN resulted in an improvement in vocal efficiency, compared with sham ovine larynges. As the absolute dimensions of larynges vary between sheep,^11^ and the volume of the TAM depends on the size of the larynx itself, the 6% increase in TAM volume of the stimulated sheep was not statistically significant given the sample size. Ideally, the volume change should be calculated from volume measurements before and after the bilateral stimulation. However, due to our micro‐CT protocol, the TAM volume cannot be determined in vivo before the start of the stimulation. Therefore, the exact increase in volume caused by FES could not be determined in the settings of this study. Nevertheless, histology showed a significant increase in minimal feret diameter of type II fibers, indicating muscle volume increase of the stimulated TAM. Similar results have been observed in resistance exercise studies,^19^, ^20^ as well as in a study using FES to stimulate a denervated PCAM.^21^ Compared with our previous study,^5^ we have modified the training pattern. One‐week stimulation every other day was chosen to support the healing process after implantation, followed by 8 weeks of stimulation every day, as studies have shown that 6–10 weeks of training promotes muscle hypertrophy.^9^, ^22^ Based on the histological analysis of fiber type distribution and MyHC gene expression analysis, this stimulation pattern, most likely, did not induce an undesired switch of the skeletal muscle fiber types. An increase in the gene expression of PPARGC1A was observed, which is often associated with a switch from fast to slow muscle fibers.^23^ Ruas et al., however, reported that there are various splice variants of the PPARGC1A (PGC‐1α), with PGC‐1α4 being highly expressed in exercised muscle but does not regulate most known PGC‐1a targets such as the mitochondrial OXPHOS genes. Rather, it specifically induces IGF1 and represses MSTN, thereby inducing robust skeletal muscle hypertrophy in mice.^24^ The RLN stimulation inevitably co‐activated the PCAM; however, the training had no adverse effect on the sole abductor of the glottis.

A number of animal studies and human trials have successfully applied FES to treat neuromuscular defects of the larynx, with the underlying pathology being VF paralysis.^21^, ^25^, ^26^, ^27^, ^28^, ^29^, ^30^ In these studies, a direct stimulation of the denervated PCAM was performed to restore the muscle function and mass. Unlike VF paralysis, where a synkinetic reinnervation alters the nerve anatomy, the nerve structure itself is intact in elderly patients.

There are, however, some limitations to this study. We have used a small sample size of only 6 animals per group. Although we could show a significant effect of the stimulation on the functional and histological levels, the long‐term effect of the stimulation has not been determined. It is likely that continuous FES stimulation will be needed to maintain the positive effect of FES, as the muscles would eventually revert to the prestimulation size after stimulation cessation, although there is some evidence that maintenance of mass may need a small amount of daily activation.^31^ Furthermore, the current procedure has been optimized for studies in large animals and is not intended for human trials. However, future minimally invasive techniques could pave the way for FES as a causal treatment for presbyphonia.

## CONCLUSION

Bilateral FES is a promising method for the causal treatment of presbyphonia by targeting the TAM. This is the first study to demonstrate the effects of FES on the molecular, histological, and functional levels. Stimulation of the terminal adductor branch of the RLN resulted in an increase of minimal muscle fiber diameter of the TAM and improved vocal efficiency of the stimulated larynges.

## Supporting information


**Data S1.** Method: “Functional analysis‐vocal efficiency.”
Method: “Triple immunofluorescence labeling of cryosections.”
**Table SI.** Primer sequences used for RT‐qPCR.
**Fig. S1.** Effect of FES on the PCAM. FES had no effect on the minimum feret diameter of muscle fibers (A) or fiber type distribution (B). RT‐qPCR showed no effect on the gene expression: Myosin heavy chain (MyHC) isoform percentages (C), PPARGC1A: peroxisome proliferator‐activated receptor gamma coactivator 1‐alpha (D), TFAM: mitochondrial transcription factor A (E), MSTN: myostatin (F) and IGF1: insulin‐like growth factor 1 (G). Data are represented as mean with standard error (A) and median with interquartile range (B–G).
