# Supplementary material for: Bilateral Functional Electrical Stimulation for the Treatment of Presbyphonia in a Sheep Model
Source: Laryngoscope. 2023 Aug 19;134(2):848–54. doi: 10.1002/lary.30984 (PMC10952233; doi:10.1002/lary.30984)
Supplement: Supplementary file 1 — Data S1. Method: “Functional analysis‐vocal efficiency.” Method: “Triple immunofluorescence labeling of cryosections.” Table SI. Primer sequences used for RT‐qPCR. Fig. S1. Effect of FES on the PCAM. FES had no effect on the minimum feret diameter of muscle fibers (A) or fiber type distribution (B). RT‐qPCR showed no effect on the gene expression: Myosin heavy chain (MyHC) isoform percentages (C), PPARGC1A: peroxisome proliferator‐activated receptor gamma coactivator 1‐alpha (D), TFAM: mitochondrial transcription factor A (E), MSTN: myostatin (F) and IGF1: insulin‐like growth factor 1 (G). Data are represented as mean with standard error (A) and median with interquartile range (B–G). [file LARY-134-848-s001.docx]

**Supplemental Information**

Functional analysis-vocal efficiency

To determine the impact of FES on the phonation, the Vocal efficiency (VE) parameter of the stimulated and sham group larynges was evaluated based on *ex vivo* experiments. The VE is a measure of the conversion efficiency of radiated acoustic power ($P_{r}$) over aerodynamic power ($P_{a}$) as following ^32^ ^33^:

$$\frac{P_{r}}{P_{a}}= \frac{4\pi r^{2}I_{0} \times{10}^{(SPL/10)}}{P_{sub} Q}$$

Where *r* is the distance between the vocal folds (VFs) and the microphone (0.3 m), $I_{0}$is the standard reference intensity (${10}^{-12}$ W/m^2^), SPL is the Sound Pressure Level, $P_{sub}$ is the subglottal pressure and *Q* is the flow rate. The subglottal pressure was measured with a XCS-93-5PSISG Kulite pressure sensor (Kulite Semiconductor Products, Inc., Leonia, NJ, USA), situated in a hole in the artificial trachea 130 mm below the glottis. The signal of the pressure sensor was directly captured by a PXIe-4330 bridge module (National Instruments, Austin, TX) at a sampling frequency of 96 kHz. For all larynges, the subglottal pressure signal, the acoustic pressure signal and the high-speed video recording of the VF oscillations were captured synchronously for different phonatory conditions. For each larynx, four different levels of pre-stress force were applied by attaching weights (20 g, 40 g, 60 g, and 80 g) to the thyroid cartilage, to emulate different phonatory conditions. The phonation onset was determined for each pre-stress force level by gradually increasing the glottal airflow until sustained phonation was achieved. Then the airflow was increased for 15 times by 5 standard liters/minute (SLM). This resulted in 768 measurement runs in total (16 runs × 4 weights × 6 larynges × 2 groups). These conditions were generated by applying different pre-stress levels in VFs and flow rate levels according to previous studies. ^11^ ^12^

Triple immunofluorescence labeling of cryosections

Cryosections (10 μm) were prepared from frozen muscle samples and correct orientation of specimens was confirmed via standard haematoxylin and eosin staining. Subsequently, sections on Superfrost Plus microscope slides (Thermo Scientific, Waltham, MA, USA) were air dried for 30min. at room temperature before fixation with methanol/acetone (1:1) at -20°C for 15min. Slides were then rinsed three times for 5min in phosphate-buffered saline (PBS) before incubation with rabbit anti-collagen 6 antibody (Abcam, Cambridge, UK) at a dilution of 1:400 for 1h. After another rinsing step as described above, Alexafluor 488 goat anti rabbit diluted 1:1000 was added for 1h followed by a further rinsing step. Slides were then exposed to two different mouse monoclonal antibodies: (i) anti-type 1 myosin heavy chain NOQ7.5.4D (Merk Millipore Corp., UK) directly labelled with Zenon 350 (Fisher scientific, UK) diluted 1:50, (ii) anti-type 2 myosin heavy chain MY32 (Abcam, Cambridge, UK) directly labelled with Zenon 594 diluted 1:1000. After incubation for 1h and washing as described above, sections were post fixed using 4% paraformaldehyde in PBS (15 min) followed by a final PBS washing step and mounting in Vectashield mounting medium (Vector Laboratories, Peterborough, Cambridgeshire, UK). The sections were examined using a fluorescence microscope (Leica DM4000B microscope) with filters designed for each of the emitting wavelengths used. Slides were examined using a x10 objective and representative sections were selected by an experienced observer. These were chosen based on the presence of minimal artefacts, optimal sectioning of the muscle fibers, and relatively uniform tissue appearance. For each muscle, two representative images were captured using Zen 2.6 (blue edition) software (Leica, Jena, Germany) and exported in.czi format to Muscle classification tool v1.3 software (Trier University of Applied Sciences^34^). Background correction was performed using the blank images collected with the same objective and each filter block used and images converted into grey level .tiff format ready for image analysis. The software then made a count of the fibers and measured the minimum feret diameter for each using the collagen grey level of each image. The system was calibrated for the microscope and x10 objective used by measuring a slide graticule (1pixel = 1.01μm).

Supplemental Table S1. Primer sequences used for RT-qPCR

| Gene Symbol | Also Known As | NCBI Reference Sequence | Forward Primer | Reverse Primer |
| --- | --- | --- | --- | --- |
| B2M |  | NM_001009284.2 | GCCATCCAGCGTATTCCAGA | CCCCGTTCTTCAGCAAATCG |
| UXT |  | XM_012183356.1 | GACTCCAGGAAGCTAATCATCC | TGAGAGCTTCTGCCAGTGT |
| LOC442994 | MYH1 | XM_004012706.3 | GTTCTCTGGCGCAGCATCT | GAGTGCTCCTCAGGTTGGTC |
| LOC443471 | OMYHC2A, MYH2 | XM_015098654.1 | AATGGCAGTCTTTGGGGAGG | AAAGATTCCTTGGGCTCGGC |
| LOC101111980 | MYH4 | XM_004012702.2 | CTGCAAGACTTGGTGGACAA | TGGAGTTTGCGGAATTTGGA |
| MYH7 | OMYHCS | XM_004010325.2 | TGCTGACAGACAGAGAAAACCAG | TTTTGCTGCGGTCGCCAAT |
| LOC101103165 | MYH13 | XM_012185735.2 | TGCAGAAGCAGGCGACTC | TTTCCCTGAACACAGCGGAC |
| MSTN |  | NM_001009428.3 | TGCCCACGGAGTCTGATCTT | CCACAGTTGGGCCTTTACTACT |
| PPARGC1A | PGC1, PPARGC1 | XM_012179733.2 | AAGGCAATTGAAGAGCGCCG | AGCTGTCTCCATCATCCCGC |
| TFAM |  | XM_015104510.1 | AGCTCAAAACCCAGATGCAAAA | TATACCTGCCAGTCTGCCCT |
| IGF1 |  | NM_001009774.3 | GAGACAGGGGCTTTTATTTCAACA | TCCAGCCTCCTCAGATCACA |


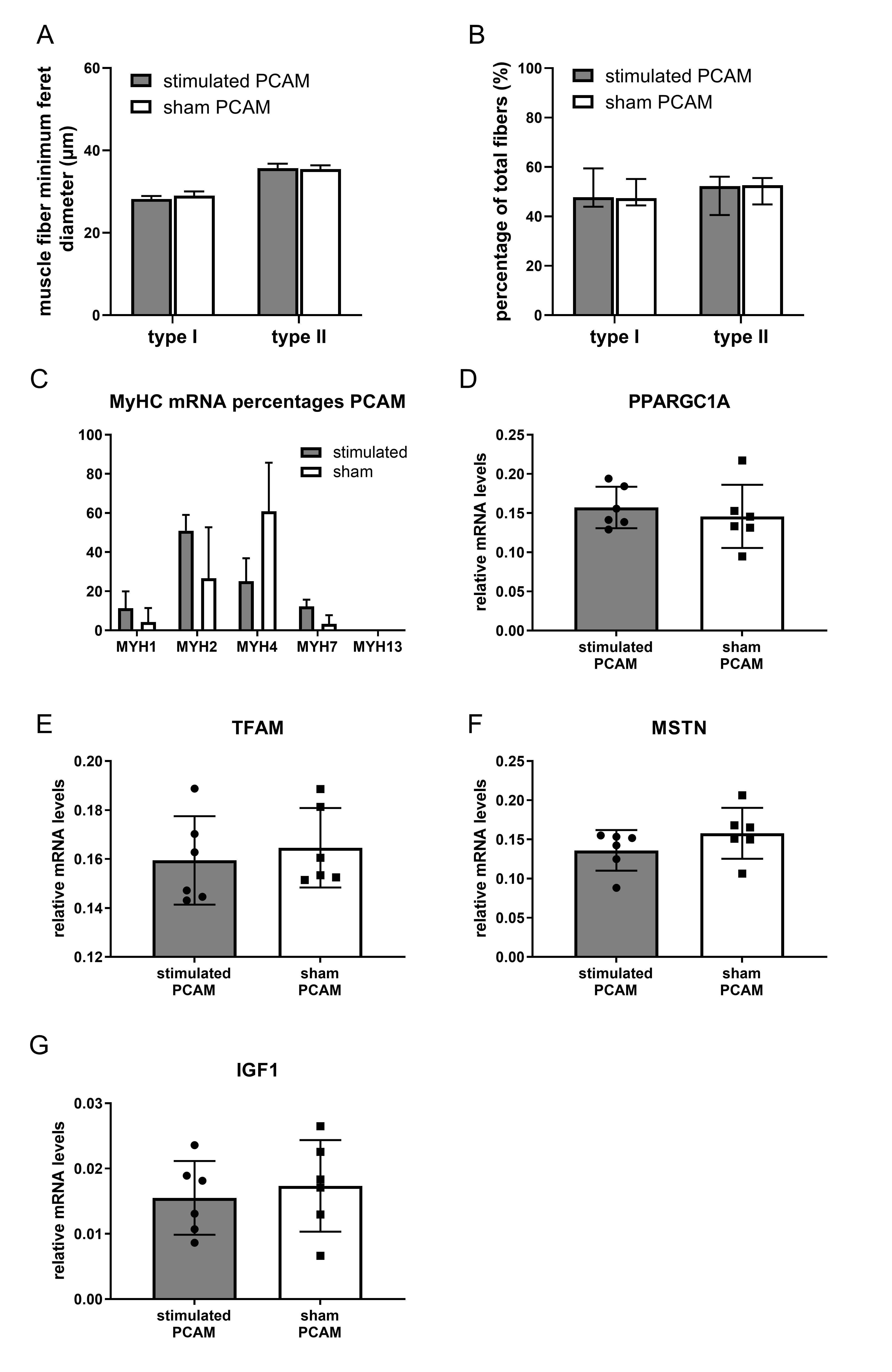


Supplemental Figure S1. Effect of FES on the PCAM. FES had no effect on the minimum feret diameter of muscle fibers (A) or fiber type distribution (B). RT-qPCR showed no effect on the gene expression: Myosin heavy chain (MyHC) isoform percentages (C), PPARGC1A: Peroxisome proliferator-activated receptor gamma coactivator 1-alpha (D), TFAM: Mitochondrial transcription factor A (E), MSTN: Myostatin (F) and IGF1: Insulin-like growth factor 1 (G). Data are represented as mean with standard error (A) and median with interquartile range (B-G).
